# Comprehensive analysis of Cuproplasia and immune microenvironment in lung adenocarcinoma

**DOI:** 10.3389/fphar.2023.1240736

**Published:** 2023-09-15

**Authors:** Junjie Kuang, Zemao Zheng, Wen Ma, Shaohui Zeng, Dehua Wu, Xie Weng, Yuming Chen

**Affiliations:** ^1^ Dongguan Institute of Clinical Cancer Research, Dongguan Key Laboratory of Precision Diagnosis and Treatment for Tumors, Affiliated Dongguan Hospital, Southern Medical University, Dongguan, Guangdong, China; ^2^ Department of Respiratory and Critical Care Medicine, Nanfang Hospital, Southern Medical University, Guangzhou, Guangdong, China; ^3^ Department of Medical Oncology, Affiliated Cancer Hospital and Institute of Guangzhou Medical University, Guangzhou, Guangdong, China; ^4^ Shenzhen Hospital (Futian) of Guangzhou University of Chinese Medicine, Shenzhen, Guangdong, China; ^5^ Department of Radiation Oncology, Nanfang Hospital, Southern Medical University, Guangzhou, Guangdong, China; ^6^ Cancer Center, Integrated Hospital of Traditional Chinese Medicine, Southern Medicine University, Guangzhou, Guangdong, China

**Keywords:** Cuproplasia, lung adenocarcinoma (LUAD), prognostic models, gene signatures, immunotherapy

## Abstract

**Background:** Trace elements such as copper are essential for human health. Recently the journal Nat Rev Cancer has put forward the concept of Cuproplasia, a way of promoting tumor growth through reliance on copper. We attempted to conduct a comprehensive analysis of Cuproplasia-related genes in lung adenocarcinoma (LUAD) to explore the mechanism of action of Cuproplasia-related genes in LUAD.

**Method:** Transcriptome data and clinical information of LUAD were obtained from TCGA-LUAD and GSE31210, and prognostic models of Cuproplasia-related genes were constructed and verified by regression analysis of GSVA, WGCNA, univariate COX and lasso. The signal pathways affected by Cuproplasia-related genes were analyzed by GO, KEGG and hallmarK pathway enrichment methods. Five immunocell infiltration algorithms and IMVIGOR210 data were used to analyze immune cell content and immunotherapy outcomes in the high-low risk group.

**Results:** In the results of WGCNA, BROWN and TURQUOISE were identified as modules closely related to Cuproplasia score. In the end, lasso regression analysis established a Cuproplasia-related signature (CRS) based on 24 genes, and the prognosis of high-risk populations was worse in TCGA-LUAD and GSE31210 datasets. The enrichment analysis showed that copper proliferation was mainly through chromosome, cell cycle, dna replication, g2m checkpoint and other pathways. Immunoinfiltration analysis showed that there were differences in the content of macrophages among the four algorithms. And IMVIGOR210 found that the lower the score, the more effective the immunotherapy was.

**Conclusion:** The Cuproplasia related gene can be used to predict the prognosis and immunotherapy outcome of LUAD patients, and may exert its effect by affecting chromosome-related pathways and macrophages.

## Introduction

There are currently more deaths from lung cancer than any other type of cancer worldwide (Bray et al., 2018). Lung adenocarcinoma (LUAD) is the most common pathological type of lung cancer, and the incidence of LUAD is increasing year by year. They tend to be younger, with fewer initial symptoms and more rapid onset, The fatality rate is high and the prognosis is poor. Most patients are diagnosed late period (Hong et al., 2020).

The human body contains trace elements, which constitute a minute fraction, 0.005%–0.01%, of its total composition, including iron, zinc, nickel, copper, selenium, iodine, manganese, cobalt, chromium, vanadium, fluorine, silicon, molybdenum, and tin (Costa et al., 2023; Frydrych et al., 2023; Xie et al., 2023; Yang et al., 2023). Despite their minimal content, trace elements are crucial for human health as they perform essential physiological and biochemical functions. Recent studies have revealed a significant association between trace elements and tumor progression and mortality (Aishajiang et al., 2023). One element of particular interest is copper, the level of which is critical for tumor development (van Renterghem et al., 2023). Both tumor tissue and serum copper levels in cancer patients are significantly elevated, with increased copper concentrations observed in a variety of tumors, including breast, prostate, lymphoma, cervical, lung, and stomach cancers (Denoyer et al., 2015).

Clinical evidence further underscores the importance of copper in cancer development. Specifically, abnormal copper accumulation in Wilson disease patients fosters malignant transformation of liver cells (Jopowicz and Tarnacka, 2023). Moreover, the Mitogen-activated protein kinases (MAPK) signaling pathways, which are intimately involved in the development of various cancers (Turski et al., 2012), are influenced by copper levels. Approximately 40%–50% of melanomas and other common tumors, such as lung, thyroid, and colorectal cancers, exhibit BRAF gene mutations, resulting in the constitutive activation of MAPK channels (Dankner et al., 2018). Dankner et al. (2018) discovered that intracellular copper directly binds and activates MAPK kinase (MEK), promoting MAPK signaling in *Drosophila melanogaster* and consequently leading to tumor development. Additionally, Brady DC et al. (Brady et al., 2014) demonstrated that copper ions enhance MEK phosphorylation of downstream extracellular signal-regulated kinase (ERK) through MEK interaction. Decreasing intracellular Ctr1 expression inhibits BRAF-mediated downstream ERK signaling pathway activation. Similarly, mutating the MEK1 copper binding site achieves the same inhibitory effect. Interestingly, Tsang T et al. (Tsang et al., 2020) identified MeK1-like copper binding sequences in unc-51-like kinase 1 (ULK1) and unc-51-like kinase 2 (ULK2). Further experiments confirmed that copper ions binding to ULK1 or ULK2 activates them, stimulating the autophagy pathway and resulting in mouse lung cancer proliferation.

The influence of copper ions extends to other signaling pathways as well. Some studies have demonstrated that copper ions bind to 3-phosphoinositide-dependent protein kinase-1 (PDK1), enhancing its interaction with serine/threonine protein kinase AKT (also known as protein kinase B) and activating AKT’s oncogenic signaling in a phosphatidylinositol-3-kinase (PI3K)-dependent manner. Inhibiting the copper axis diminishes AKT signaling and suppresses tumor development, indicating a close relationship between the PI3K-PDK1-AKT axis and tumor proliferation (Guo et al., 2021).

Recently, the concept of Cuproplasia was first proposed in the journal nature reviews cancer to try to explain the cell proliferation mode dependent on copper ions. In this study, we carried out landscape description of the Cuproplasia genes summarized in this review in LUAD. The Cuproplasia risk score (CRS) was established by Least absolute shrinkage and selection operator (LASSO) regression analysis to predict the overall survival (OS) of LUAD patients, and multiple omics analysis was used to try to explain the specific role of Cuproplasia.

## Materials and methods

### Data collection and collation

In The Cancer Genome Atlas-lung adenocarcinoma (TCGA-LUAD) database, data categories were selected as transcrip‐tome profiling and raw counts, including 535 primary lung adenocarcinoma samples and 59 normal samples. Clinical information including complete gender, age, survival time, survival status, and pathological stage of 201 cases was downloaded from UCSC for subsequent analysis. Transcriptome data and clinical information of 210 patients in GSE31210 were downloaded from the (Gene Expression Omnibus) GEO database. R software is used to process the data sets of TCGA and GEO. Gene expression levels are determined by the average value of expression if multiple probes correspond to the same gene. The Benjamini–Hochberg method is adopted to adjust the *p* value to control the false discovery rate (FDR). After removing duplicate genes and their expression in the original data downloaded from the TCGA database, the CPM function of the R software edgeR package was used to correct and standardize the data, and the mean CPM (count value of each transcript per million bases) ≤1 was deleted, and then we take the logarithm base 2. The copper proliferation gene was obtained from the literature (Ge et al., 2022).

### Gene set variation analysis (GSVA) scores of Cuproplasia genes

GSVA is a method that can be applied to Microarray and RNA-sequence data sets of enrichment under the conditions of no parameters and without supervision by a trained scientist (Hänzelmann et al., 2013). GSVA can convert a gene-sample data matrix into gene-set - sample matrix. Based on this matrix, the enrichment of gene sets (such as KEGG pathway) in each sample can be further analyzed. Since GSVA is a gene-sample Enrichment matrix, downstream Analysis would be allowed more freedom than other Gene Set enrichment methods, such as Gene Set Enrichment Analysis (GSEA) (Yi et al., 2020). In this study, we scored the copper proliferation level of patients in TCGA-LUAD based on the Cuproplasia-related genes.

### Weighted gene co-expression network analysis (WGCNA)

From whole-genome expression analysis, WGCNA provides valuable information about gene function and gene association, and can be used to detect module-membership (MM) of highly correlated genes and a module related to gene-significance (GS) that provides insight into the function of co-expressed genes, it can also assist in the identification of genes that play a critical role in the development of human diseases (Zhang and Horvath, 2005; Saris et al., 2009; Li et al., 2022; Cheng et al., 2023). The co-expression network was constructed by using the WGCNA package of R software to construct the TCGA data set. The Pearson coefficient between each gene was firstly calculated to convert it into a similar matrix, and the soft threshold β was automatically selected for network topology analysis through the pick soft threshold function of the WGCNA package. β can emphasize the strong and weak correlation between genes (Langfelder and Horvath, 2008) and β is set to 4, scale-free = 0.9. After β was determined, the similar matrix was transformed into an adjacency matrix, and then the adjacency matrix was transformed into a topological overlap matrix (TOM). The minimum number of genes in the module was set as 50, and the shear height was set as 0.25. The genes with similar expressions were placed in the same gene module through hierarchical clustering, and the threshold was set as 20,000 to eliminate outliers. Genes that express similar patterns are grouped into different modules. Gene modules closely related to tumorigenesis were selected based on correlation coefficients between genes and phenotypes (cancer tissue and normal samples). If a gene in the module has both large MM and GS, it is considered to be the core gene in the module, and the MM > 0.7 and GS > 0.35 was defined as the candidate core gene. Then, intersection of central genes selected by Cytohubba and core genes selected by modules was selected, and genes in the intersection were defined as the final key genes.

### Acquisition of differential genes in cancer tissue and normal tissue

A powerful transcriptomics technique is differential gene expression analysis, which demonstrates quantitative changes in gene expression between normal and cancer cells based on molecular mechanisms. Such differences in gene expression can reveal potential biomarkers for specific diseases. We use R software packages for key genes Limma for differences in gene screening (Ritchie et al., 2015), selection criteria for: | log2 (fold—change) | ≥1 and the corrected *p* value (false discovery rate, FDR) ≤ 0.05.

### Screening of prognostic related copper proliferating genes

Univariate Cox regression analysis was performed for differential gene expression and overall survival between tumor and normal tissues to screen out prognostic copper proliferating genes. The screening criteria were *p* value less than 0.05 (Lunn and McNeil, 1995).

### LASSO regression analysis

The Least Absolute Shrinkage and Selection Operator (LASSO) is a regression analysis method that performs both variable selection and regularization in order to enhance the prediction accuracy and interpretability of the statistical model it produces (Li et al., 2023; Tu et al., 2023). By using the glmnet package, survival status was used as the dependent variable, and the expression value of the selected differential prognostic genes was used as the response variable. 1,000 Lasso regression analyses were carried out to reduce the number of genes, so as to reduce the error of the model and obtain a generalized linear model (Tibshirani, 1997). The Prognostic genes were identified using multivariate Cox proportional risk regression analysis. Predicting prognosis status was based on CRS. Molecular expressions of individual genes in the sample were taken into account in determining the multivariate Cox proportional risk regression scores for each patient. Following is a detailed description of the calculation formula:

$$CRS=\sum_{i=1}^{n}{\mathrm{Exp}}_{i}*{Coef}_{i}$$

### Receiver operating curve (ROC curve) of the relationship between CRS and prognosis

We calculated the CRS for the training dataset (TCGA-LUAD) and validation (GSE31210) using the prognosis model, and we divided the validation and training sets by the median CRS. The survival curve was drawn based on the survival information to obtain the survival status of high and low risk expression, and the prediction effect of the model was evaluated (≤0.05), the statistical method used in this process is the log-rank test. To evaluate regression models’ predictive ability in 1-year, 3-year, and 5-year survival, the time-dependent ROC curve was calculated using R software’s “survival ROC” package. When the AUC is larger than 0.5 and closer to 1, the prediction effect is better.

### Functional enrichment analysis

To explore the pathogenesis and development mechanism of lung adenocarcinoma, gene function analysis (gene ontology, GO) and pathway analysis (Kyoto encyclopedia of genes and genomes, Genomes, KEGG) for detailed biological annotation and description of the function of gene products. GO covers molecular function (MF), cellular components (CC) and biological processes (BP). The functional information of a given gene was comprehensively summarized through enrichment analysis (Harris et al., 2004). KEGG incorporates information about genomics, chemical processes, and systematic functions. The method analyzes gene function from all angles of gene and molecular networks, which are thought to be responsible for identifying metabolic and functional pathways (Kanehisa and Goto, 2000). The path enrichment analysis was also carried out by hallmark.

### Relationship between GSVA score and riskscore

Spearman method was used to analyze the correlation between GSVA score and riskScore of Cuproplasia gene set.

### Immune checkpoint analysis

The expression levels of 10 common immune checkpoints (CTLA4, PDCD1, CD274, ICOS, LAG3, BTLA, TNFRSF14, NRP1, CD28, and CD44) were analyzed among high and low risk groups in the TCGA-LUAD cohort to determine whether the CRS could be used in the treatment of immune checkpoints.

### Relationship between CRS and immunotherapy

Scoring and immunotherapy were performed using the IMVIGOR210 CoreBiologies. We downloaded the data of TCGA-LUAD patients from this dataset for analysis. The specific method of analysis was to compare immunotherapy outcomes in low-risk patients, including complete response (CR), partial response (PR), stable disease (SD) and progressive disease (PD).

### Hierarchical analysis

To observe the effectiveness of riskScore in different clinical stages, LUAD patients were divided into four stages according to the American Joint Committee on Cancer staging (AJCC) staging method, and K-M survival curves of high and low risk groups were drawn in each stage according to the median CRS.

### Analysis of immune cell infiltration

Immune cell infiltration and risk scoreUtilizing the xCell method (Aran et al., 2017), we assessed the enrichment levels of 64 immunological markers to evaluate the immune cell invading the microenvironment. Using techniques including CIBERSORTx (Steen et al., 2020), ssGSEA (Yi et al., 2020), quanTIseq (Finotello et al., 2019), TIMER, and MCPcell from the R package immunedeconv version 2.0.4, we conducted more exhaustive analyzes.

### Cell culture

The human LUAD cell line A549 was purchased from the American Type Culture Collection (ATCC) and cultured in RPMI 1640 medium supplemented with 10% fetal bovine serum (FBS) and 105 IU/L penicillin and 0.1 g/L streptomycin, at 37°C in a humidified atmosphere containing 5% CO2.

### siRNA transfection

Lipofectamine 2000 was used for transient transfection of siRNA. Cells were plated in advance and A549 cells in logarithmic growth phase were adjusted to a density of 1 × 10^5 cells/mL. When the confluence reached 60%, the siRNA complexes were added to the plated cells along with the transfection reagent in serum-free medium, and after 6 h, the medium was replaced with serum-containing medium. 48 h post-transfection, RNA or protein was extracted to assess transfection efficiency.

### RT-qPCR

Cells were seeded in 6-well plates and transfected with si-LAG3 for 48 h, then the medium was discarded and cells were collected. Total RNA was extracted using the Trizol one-step method. The reaction system was prepared according to the instructions of the kit, and mRNA was reverse transcribed to cDNA at 37°C for 60 min. The PCR reaction system was prepared with 2.5×RealMaster Mix/20×SYBR solution 4.5 μL, 1 μL of each primer, 2 μL cDNA, and 1.5 μL of triple-distilled water, making up a total of 10 μL. The primer sequences are shown in Supplementary Table S1. The reaction conditions were 94°C for 15 min, followed by 40 cycles of 94°C for 20 s, 56°C for 30 s, and 68°C for 30 s. GAPDH was used as an internal control to calculate the expression of the target RNA. The primer sequence for si-LAG3 is as follows: GGA​GAC​AAU​GGC​GAC​UUU​A (5’-3’).

### Western blot

Cells were seeded in 6-well plates and transfected with si-LAG3 for 48 h. The medium was then discarded, and the cells were collected and lysed on ice for 30 min. After centrifugation, the supernatant was collected, and protein concentration was determined using a BCA protein assay kit. The proteins were then separated by SDS/PAGE electrophoresis and transferred onto PVDF membranes. The membranes were blocked, incubated with primary antibodies overnight at 4°C, then with secondary antibodies for 1 h at room temperature, and finally, the bands were visualized using an ECL detection kit.

### CCK8 assay

Cells in logarithmic growth phase were prepared into a cell suspension at a density of 5 × 10^3 cells/mL, and 100 µL of the cell suspension was seeded into each well of a 96-well plate and incubated. At 1d, 2d, 3d, and 4d post-seeding, 10 µL of CCK-8 solution was added to each well and incubated for 1–2 h. The absorbance at 450.0 nm was measured using a microplate reader.

### Transwell cell migration assay

Adherent cells were digested and resuspended in serum-free medium. The cell suspension was counted and diluted to a density of 2 × 10^6 cells/mL with serum-free medium. A pipette was used to mix the cell suspension thoroughly. Transwell chambers with 8.0 µm pores were placed into a 24-well plate, and 400 µL of the cell suspension was added to the upper chamber, while the lower chamber was filled with medium containing 20% FBS. The plate was incubated at 37°C. After 36 h, the chambers were removed, and the cells were fixed with 4% paraformaldehyde for 20 min. The paraformaldehyde was then washed off with ultrapure water, and the cells were stained with crystal violet for 5 min. Excess stain was washed off with water, and the cells in the upper chamber were gently removed with a cotton swab. The chambers were observed under a microscope, and images were captured from five different fields of view. The cells were counted using ImageJ software.

### Wound healing assay

Logarithmically growing cells were resuspended in culture medium and seeded into 6-well plates. When the cell density reached 90%, a straight line was scratched in the center of each well using a 200 µL plastic pipette tip. The scratched cells and debris were washed off with PBS, and serum-free medium was added to each well. The plate was incubated at 37°C, and images of the wounds were captured at 0 h and 24 h. The wound area was measured using ImageJ software.

### EDU/DAPI staining for cell proliferation

After seeding and transfecting cells with siRNA, 2xEDU solution was added to an equal volume of culture medium containing the experimental cells and co-incubated for 12 h. The medium was then discarded, and the cells were fixed with 2.5 mL of PBS containing paraformaldehyde for 15 min at room temperature. The fixative was removed, and the cells were washed three times with PBS. Then, 2.5 mL of permeabilization solution was added, and the cells were incubated for 20 min at room temperature. The permeabilization buffer was removed, and the cells were washed twice with PBS. The staining reaction mixture was prepared according to the instructions of the EDU staining kit, and 100 mL of the mixture was added to each well. The cells were incubated in the dark for 30 min at room temperature, then washed once with PBS. Under a fluorescence microscope, the proliferating cells stained with EDU appeared red, while the cell nuclei stained with DAPI appeared blue.

### Statistical analysis

In comparing two categories with normal distributions and non-normally distributed data, statistical significance was determined using independent t-tests and Mann–Whitney U tests. Multiple category differences were compared using one-way analysis of variance (ANOVA) and Kruskal–Wallis analyses. The R package Hmisc 4.4.1 was employed to conduct Spearman and distance correlational analysis. Objects having a correlation coefficient greater than 0.5 were considered highly related. To determine the prognostic variables, Cox regression analysis was carried out. Prior to creating the survivorship curves using the R package survminer, the overall survival (OS) and CRS were calculated with the R package survival, and cutoff values were set with the R program survminer. Utilizing the R package Complex Heatmap 2.4.3, every heatmap was generated. Using the ggplot2 R software, data comparisons were illustrated. All statistical analyzes were performed on both sides of the data using R software. Statistical significance was determined by a *p*-value of 0.05.

## Results

### WGCNA result

Cuproplasia scores were performed for each patient in TCGA-LUAD through GSVA function in GSEA, and the scores of patients were shown in Supplementary Table S2 list.

**FIGURE 1:**
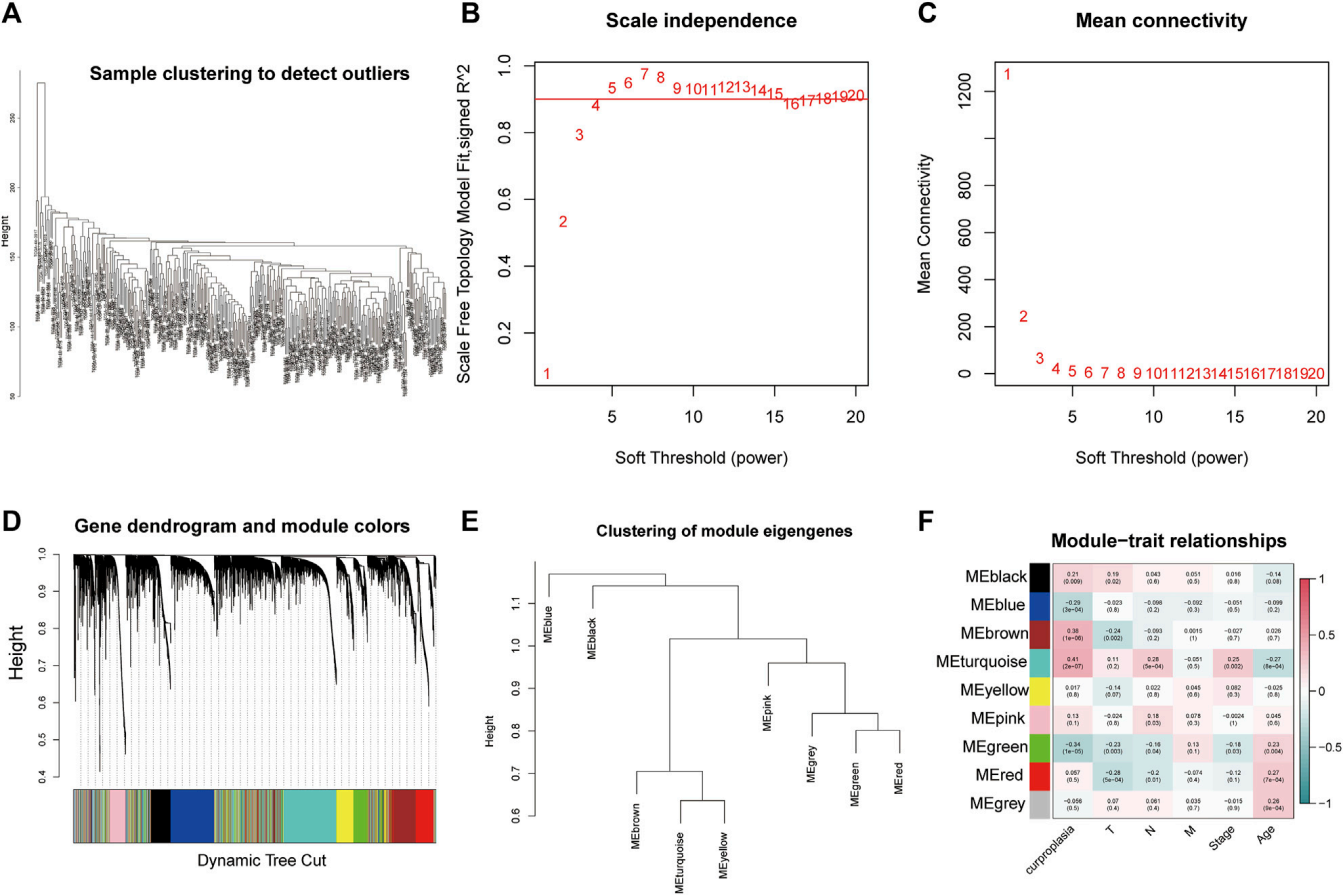
Weighted correlation network analysis results. **(A)** Sample clustering to detect outliers. **(B)** Scale independence results. **(C)** Mean connectivity. **(D)** Gene dendrogram and module colors. **(E)** Clustering of module eigengenes. **(F)** Module−trait relationships.

The WGCNA process is as follows: The gene co-expression network was established (Figures 1B,C), and the process of module identification was shown in Figures 1A,D,E. The results show that the correlation coefficients between BROWN and TURQUOISE and copper proliferation mode are 0.41 and 0.38 respectively (Figure 1F). Therefore, BROWN and TURQUOISE are considered as modules closely related to copper proliferation. There were 1,593 genes in BROWN and 3,194 genes in TURQUOISE, and the causes of 4,787 of these modules were included in subsequent analyses ().

### Construction and validation of CRS

A total of 9,064 differentially expressed genes were obtained between tumor tissues and normal tissues in the TCGA-LUAD dataset, and the results were presented in. We combined 9,064 differentially expressed genes with overall survival and performed univariate cox analysis to obtain 1,439 prognostic related genes. The intersection of 1,439 prognostic genes with the aforementioned BROWN and TURQUOISE genes obtained 214 prognostic genes related to copper proliferation.The formula of Lasso regression after dimensionality reduction is as follows: risk socres = −0.1004*PLA2G4F + 0.155*NIM1K + 0.0277*PLEK2 + 0.0402*SIRPA + 0.1713*FABP5 + 0.2555*PTX3 + 0.1306*CCT6A + 0.2177*STARD4 + 0.1917*SEC61G + −0.0174*BEX4 + 0.2549*LINC02535 + 0.2983*SHC1 + −0.269*MBOAT1 + 0.043*KRT81 + 0.0063*MT1X + −0.1954*BCAS4 + 0.0302*KRT6A + 0.0432*CD109 + −0.1846*HS3ST2 + 0.111*TINAG + 0.0055*LHX2 + 0.069*CNTNAP2 + 0.0063*KLK8 + 0.1446*CALML5。

**FIGURE 2:**
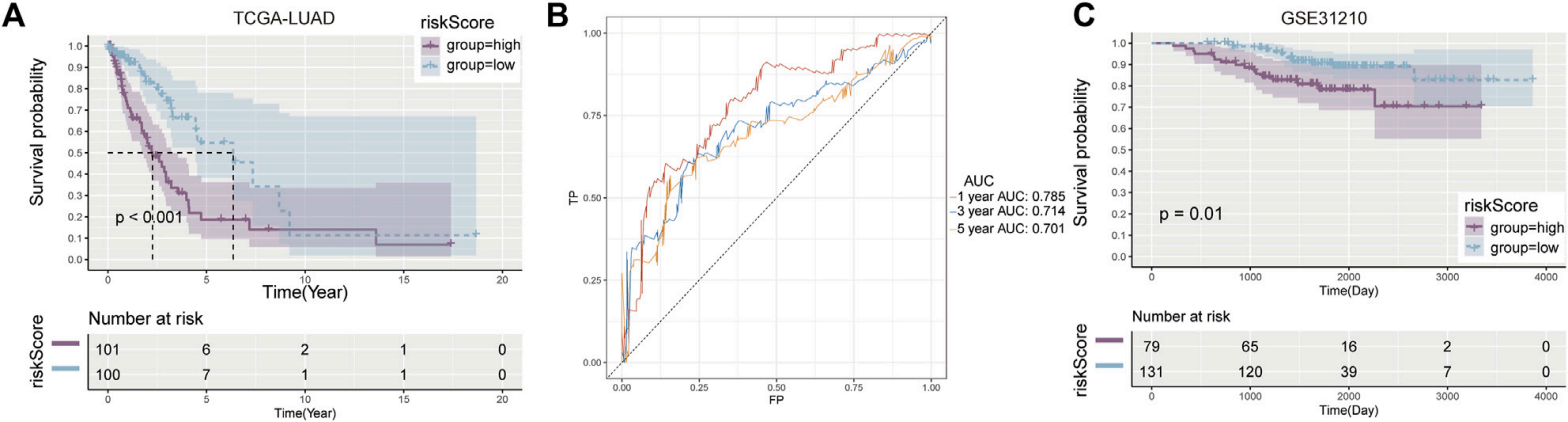
Development and validation of prognostic model. **(A)** Kaplan-Meier curves between high and low risk groups in the training cohort. **(B)** AUC values in the first, third and fifth year prognostic model. **(C)** Verify the K-M curve between high—and low-risk groups in the cohort.

According to K-M survival curves, the high risk group had a significantly higher survival rate than the low risk group (*p* ≤ 0.001) (Figure 2A). ROC curve showed that the value of AUC in the first year, the third year and the fifth year were 0.785, 0.714, and 0.701 respectively, demonstrating fair prediction ability (Figure 2B).

Similar results were observed in the validation set GSE31210, with worse survival in the high-risk group (*p* = 0.01) (Figure 2C). These results suggest that the risk score constructed by Cuproplasia-related genes can predict the OS of LUAD patients and can be used as a prognostic indicator for patients.

### Pathway of enrichment of Cuproplasia-related genes

**FIGURE 3:**
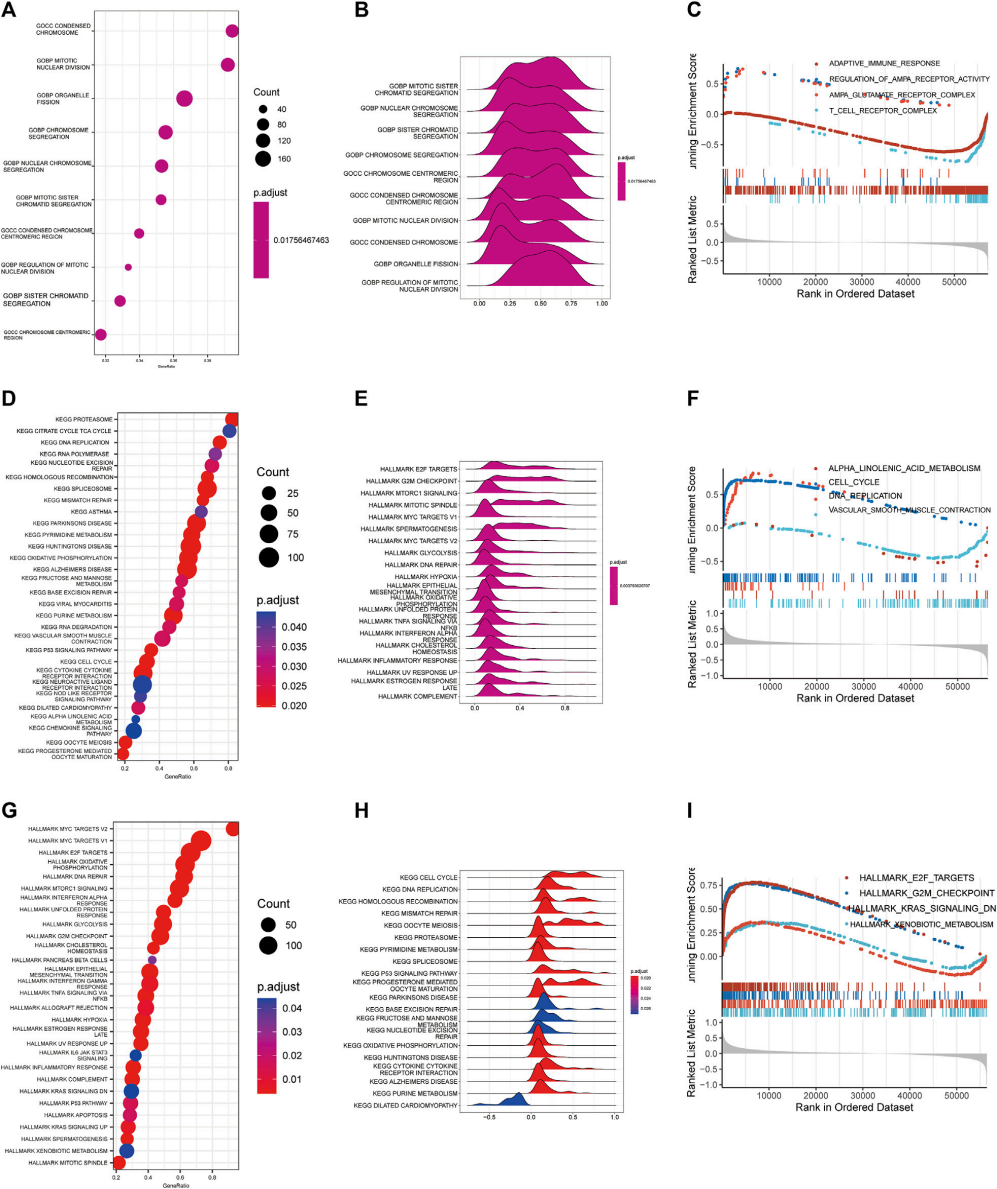
Functional enrichment analysis. Bubble diagram **(A)**, Ridge map **(B)**, GSEA diagram **(C)** of GO functional enrichment analysis. Bubble diagram **(D)**, Ridge map **(E)**, GSEA diagram **(F)** of GO functional enrichment analysis. Bubble diagram **(G)**, Ridge map **(H)**, GSEA **(I)** diagram of GO functional enrichment analysis.

GO, KEGG and Hallmark pathway enrichment methods were used to analyze the possible influence of copper proliferation-related genes on the differential genes between high and low risk groups. We showed the top ten pathways with the most significant *p* value of GO enrichment analysis, and the results were respectively enriched in mitotic sister chromatid segregation, nuclear chromosome segregation, sister chromatid segregation, chromosome segregation, chromosome centromeric region, condensed chromosome centromeric region, mitotic nuclear division, condensed chromosome, organelle fission, regulation of mitotic nuclear division. These results mainly suggest that risk scores are related to biological processes such as chromosomes. The bubble diagram is used to sort enrichment pathways by GeneRatio, and is shown in Figure 3B according to *p*-value. Figure 3C shows the four pathways with the highest NES value in the GO analysis. KEGG analysis revealed that risk scores were closely related to the following 20 pathways, and the results are shown in Figures 3 D,E. The top four pathways with the most NES score are alpha linolenic acid metabolism, cell cycle, DNA replication, vascular smooth muscle contraction (Figures 3F, G). It is suggested that Cuproplasia-related genes may play a role through these pathways and affect the OS of LUAD.HALLMARK enrichment analysis found that risk scores were closely related to the following pathways: E2F targets, G2M checkpoint, KRAS signaling down, xenobiotic meta (Figures 3H, I). LUAD’s occurrence and development may be influenced by CRS through these pathways, which affects patients’ survival chances as well.

### Risksores is closely related to immunotherapy

**FIGURE 4:**
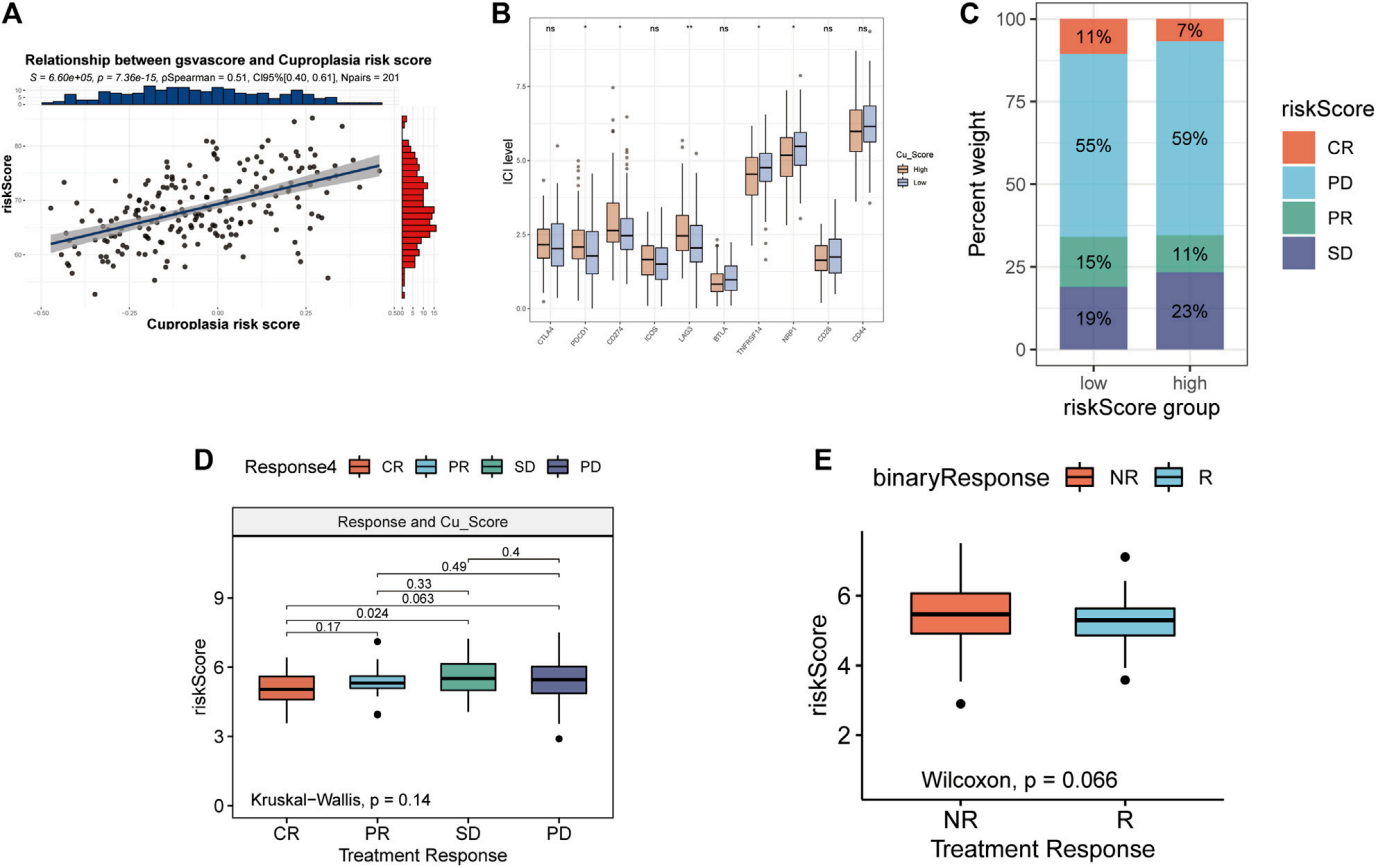
Relationship between risk scores and immunotherapy. **(A)** Relationship between gsvascore and riskScore. **(B)** The percentage of different treatment outcomes in the high-low risk group. **(C)** Expression levels of 10 immune checkpoints in different risk groups. **(D)** Risk scores in epidemic groups. **(E)** Risk scores for responders and non-responders.

The correlation between GSVA score of copper proliferation gene set and riskScore was observed, and a strong positive correlation was found (Figure 4A). Analysis of the expression levels of 10 immune checkpoints in the high-low risk group showed that PDCD1, CD274, LAG3 were highly expressed in the high-risk group, and TNFRSF14 and NRP1 were highly expressed in the low-risk group. These results suggest that CRS is a predictor of LUAD immune checkpoint therapy (Figure 4B). stack plots showed that there were more patients in the low-risk group with CR and that the difference in survival was not significant (Figure 4C). The IMVIGOR210 analysis showed that the lower the risk score, the better the treatment outcome (Figure 4D). However, binary comparisons did not show any significant differences between reactive and non-reactive groups (Figure 4E).

### Risk scores are also prognostic indicators in different clinical stages

**FIGURE 5:**
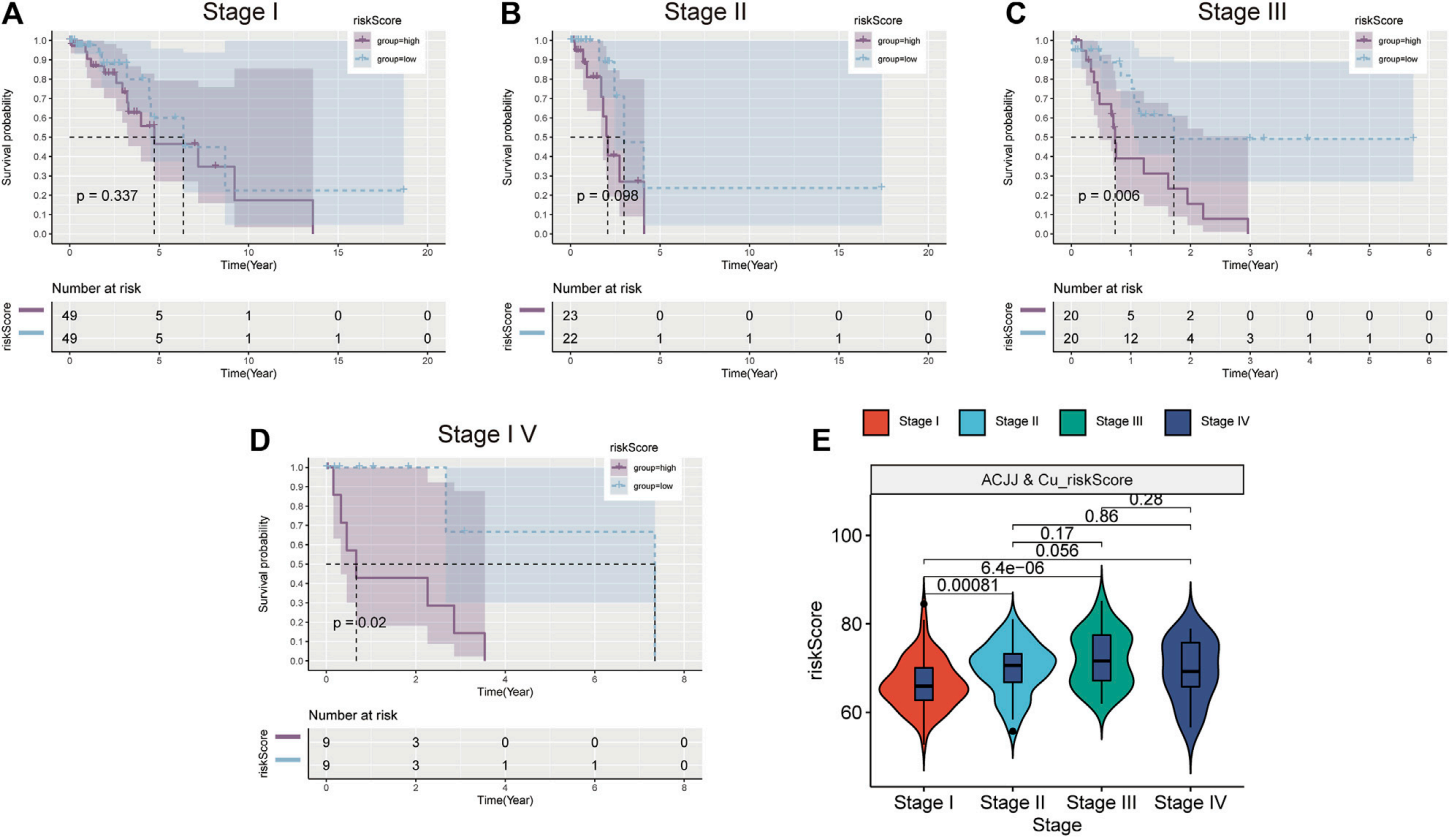
Stratified analysis based on clinical stage. K-M survival curve of high and low risk groups in Stage1 **(A)**, Stage II **(B)**, Stage III **(C)**, Stage IV **(D)**. **(E)** Comparison of risk scores in different stages.

According to the AJCC, patients at different stages were divided into high-low risk group according to the median risk score, and it was found that patients in the high-risk group of stage III and stage IV patients also had worse prognosis (Figures 5C,D). However, the high-risk group in stage I and stage II also had a worse trend (Figures 5A,B). This suggests that the higher the stage, the greater the clinical value of the risk score. Figure 5E shows the relationship between risk scores and stages, and the results show that the risk scores of stage I, stage II and stage III are different. These results indicate that risk score is closely related to clinical stage and has better clinical predictive value in stage III and IV patients.

### The relationship between risk scores and immune cells

**FIGURE 6:**
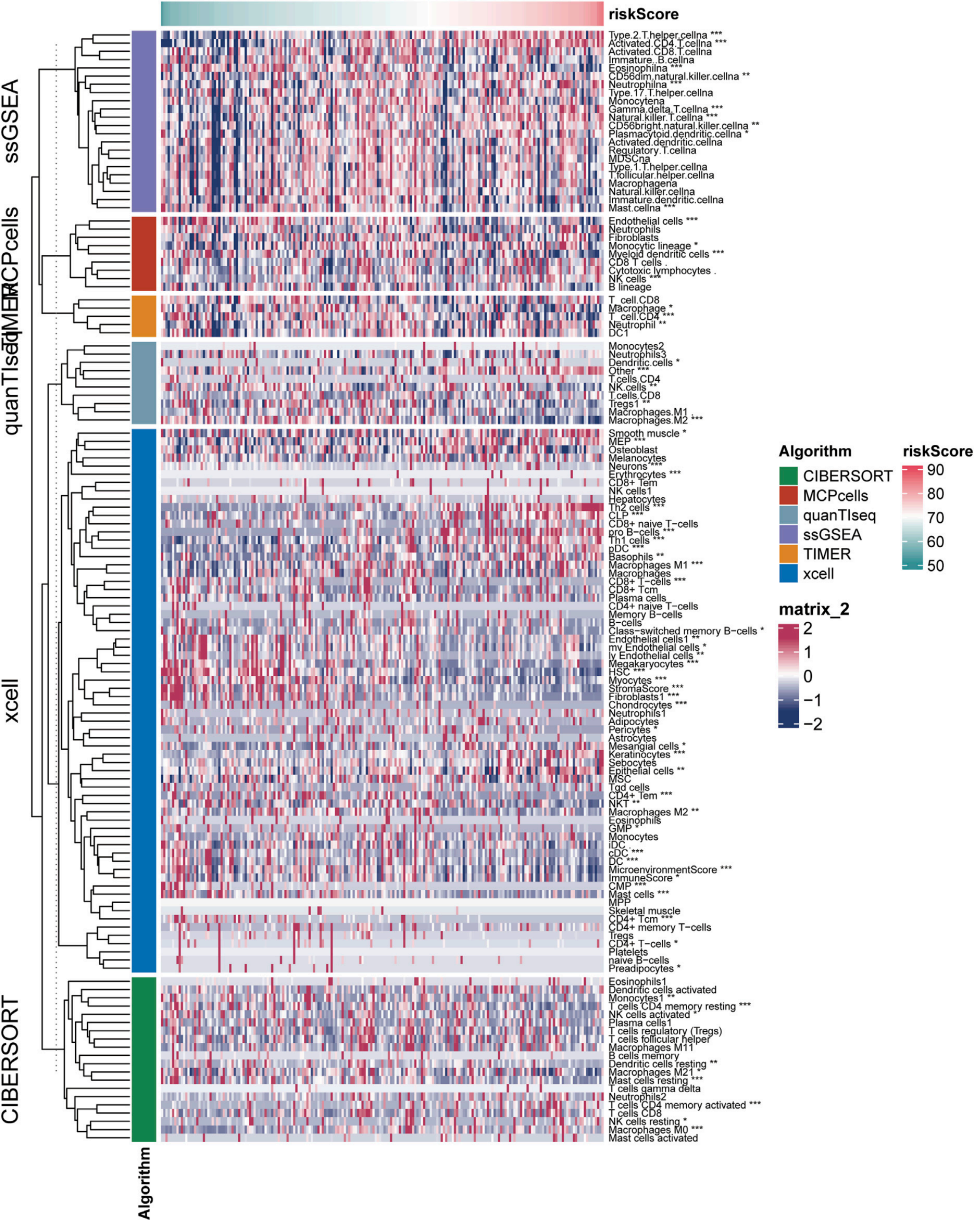
Results of six immune cell analysis algorithms in high—and low-risk groups.

**FIGURE 7:**
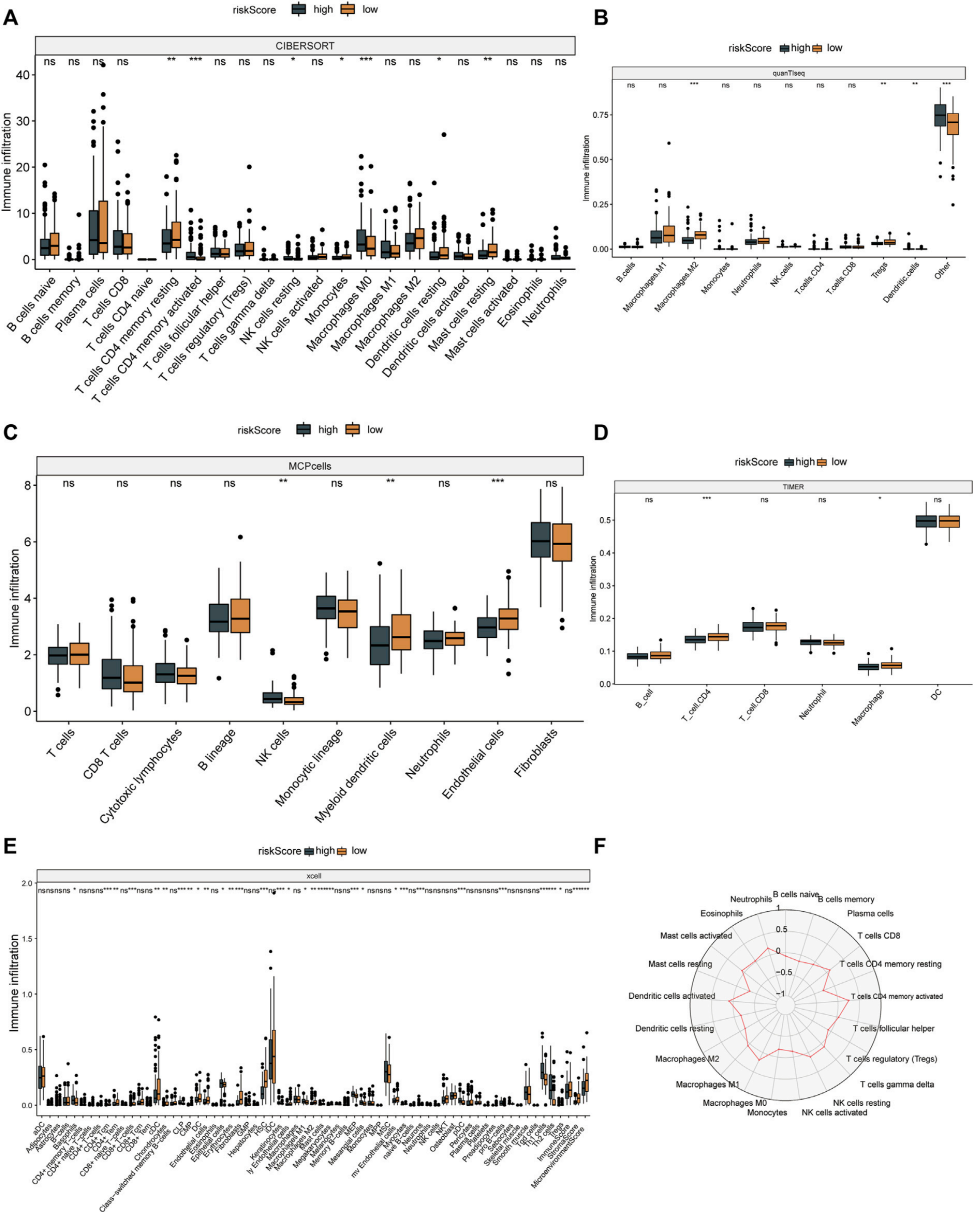
Immune cell analysis results. CIBERSORT **(A)**, quanTIseq **(B)**, mcp cells **(C)**, TIMER **(D)** and xcell **(E)** algorithms were used to analyze the immune cells in high and low risk groups. **(F)** Radar map of immune cells in relation to risk scores in CIBERSORT.

The results of six immune cell analysis algorithms between high and low risk groups are shown in Figure 6, in which immune cells with significant differences are specially labeled, and multiple types of immune cells are decreased. The analysis results of each algorithm are shown separately in Figure 7. CIBERSORT algorithm in Figure 7A shows that there are NK cells resting, T cells CD4 memory resting, T cells CD4 memory activated, Monocytes and Macrophages M0 The infiltrate contents of Dendritic cells resting and Mast cells resting were different between high and low risk groups. MCPcells showed that NK cella, Myeloid dendritic cells, and Endothelial cells were differentially expressed in high and low risk groups. quanTIseq analysis showed that the infiltrate content of acrophages.M2, Tregs and Dendritic.cell were significantly different among the high and low risk groups. CD4 T cell, Macrophage had significant difference between high and low risk groups. xcell analysis suggested significant differences in the content of a large number of immune cells between high and low risk groups. It should be noted that the content of macrophages was significantly different among the four algorithm analyses. These results suggest that copper proliferating genes may influence the development of LUAD patients through these immune cells.

### Inhibition of cell proliferation and migration by LAG3 knockdown

**FIGURE 8:**
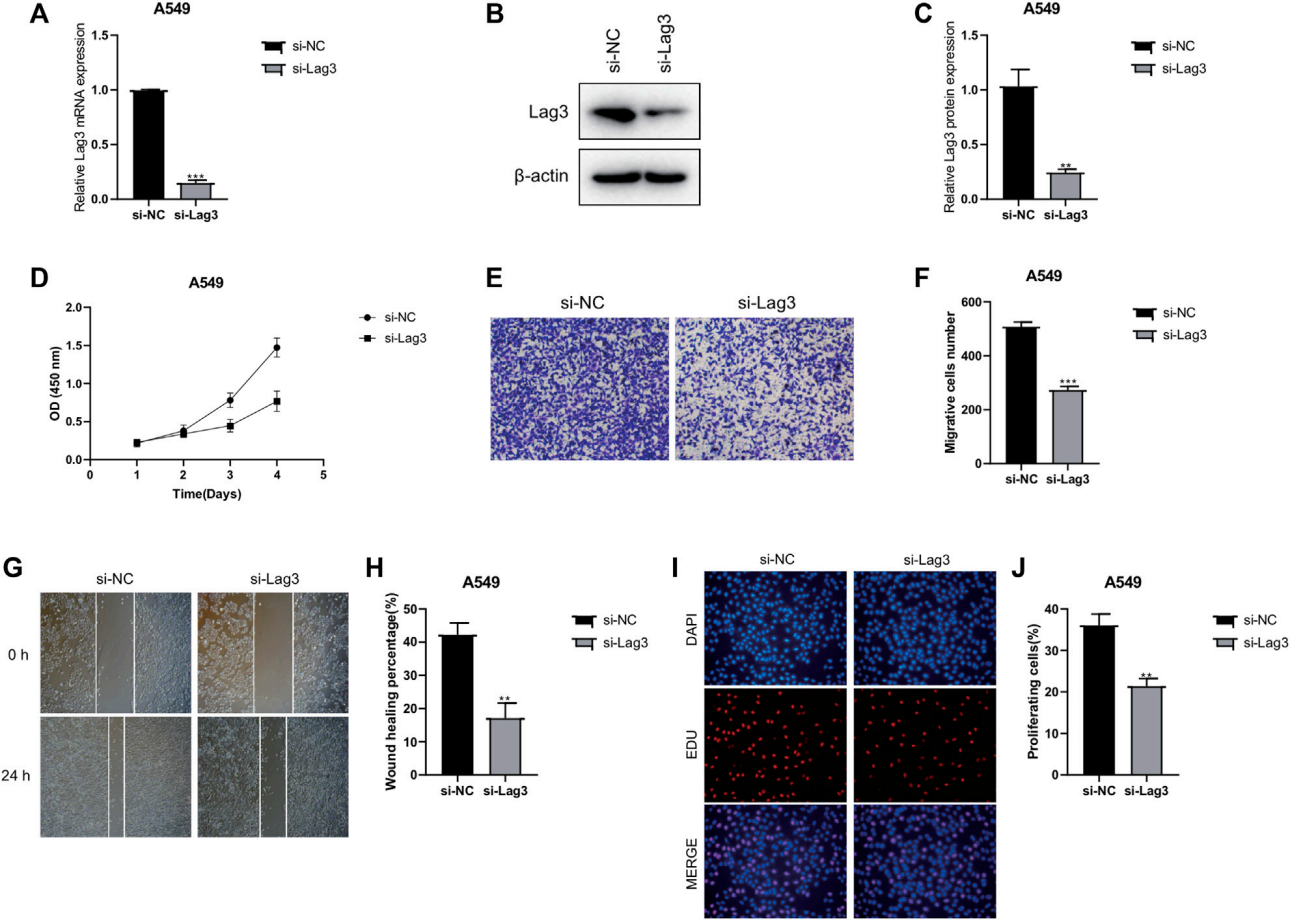
Effects of LAG3 Knockdown on A549 Cells. **(A–C)** Expression of LAG3 in A549 cells after si-LAG3 treatment assessed by qPCR and Western blot. **(D)** CCK8 assay showing the inhibition of A549 cell proliferation after LAG3 knockdown. **(E, F)** Transwell migration assay demonstrating the inhibitory effect of LAG3 suppression on A549 cell migration. **(G, H)** Scratch wound healing assay corroborating the inhibitory effect of LAG3 suppression on A549 cell migration. **(I, J)** Fluorescence microscopy images of EDU/DAPI stained A549 cells showing reduced red fluorescence intensity, indicative of inhibited cell proliferation, after si-LAG3 treatment. *p < 0.05, **p < 0.01, ***p < 0.001.

To investigate the specific mechanism of LAG3 in LUAD, we initially constructed A549 cells with stable knockdown of LAG3. The results of qPCR and Western blot experiments indicated that the expression of LAG3 in A549 cells was significantly lower after si-LAG3 treatment compared to the control group of A549 cells (Figures 8A–C). The effect of LAG3 on cell proliferation was assessed using the CCK8 assay. The results revealed that knocking down LAG3 significantly inhibited the proliferation of A549 cells (Figure 8D). Results from the Transwell cell migration assay indicated that suppressing LAG3 expression significantly inhibited the migration of A549 cells (Figures 8E,F). The scratch wound healing assay also corroborated these results (Figures 8G,H). Furthermore, we evaluated cell proliferation activity by observing cell staining under a fluorescence microscope after EDU/DAPI staining. Our experimental results showed that the intensity of red fluorescence was significantly reduced in A549 cells after si-LAG3 treatment (Figures 8I,J), suggesting that downregulating LAG3 expression in LUAD A549 cells will inhibit tumor cell proliferation.

## Discussion

Different trace elements serve distinct biological functions *in vivo*, contributing to the onset and progression of cancer to varying degrees (Cheng et al., 2019). Copper, a crucial cofactor of tyrosine kinase and ceruloplasmin, plays an indispensable role in organism growth and development. Research indicates that copper is vital for maintaining the body’s normal biological functions due to its redox properties, which enable catalysis, oxidation, cell respiration, and various other life-sustaining activities (Li, 2020). Additionally, studies have highlighted that copper ions can modulate the oxidative phosphorylation and growth processes of tumors, participate in the carcinogenic signal transduction pathway via BRAF transduction and tumorigenesis, and exhibit potential toxicity to organisms (Ishida et al., 2013; O'Leary et al., 2019; Antsotegi-Uskola et al., 2020). Hence, it is imperative to maintain the copper ion content of an organism in a relatively stable state to prevent disrupting the homeostasis of the internal environment, inducing stress responses, and causing unnecessary harm. Recently, research on cuproptosis has garnered attention. The specific mechanism involves copper ions binding to fatty acylated proteins in the mitochondrial respiration process of the TCA cycle, leading to protein aggregation. This, in turn, promotes the downregulation of Fe-S cluster proteins, inducing protein-toxic stress and ultimately resulting in cell death (Antsotegi-Uskola et al., 2020). The study of cuproptosis has piqued the interest of researchers, and some studies based on extensive transcriptome data have shed light on the mechanism of copper-induced cell death in lung adenocarcinoma (LUAD) (Wang X. et al., 2023; Wang T. et al., 2023; Ma et al., 2023). The recent evolution of perspectives on Cuproplasia may pave the way for novel avenues of research into the role of copper in tumorigenesis.

In this study, we initially identified 40 Cuproplasia-related genes and scored 313 LUAD patients based on their transcriptome using the GSVA scoring method. Through WGCNA analysis, we screened 4,787 genes in the BROWN and TURQUOISE modules, which are closely related to the Cuproplasia score. Subsequently, 214 prognostic-related genes were identified through univariate Cox analysis, and a prognostic model was established based on 24 genes through lasso regression analysis. We found that risk scores could predict overall survival (OS) in both TCGA-LUAD and GSE31210 cohorts. These results suggest that genes related to copper proliferation play a significant role in the prognosis of LUAD patients. Among the genes included in the prognostic model, PLEK2 has been reported in five studies as a prognostic marker for lung adenocarcinoma (Cheng et al., 2019; Li, 2020; Ishida et al., 2013; O'Leary et al., 2019; Antsotegi-Uskola et al., 2020). Cell invasion, cell cycle, DNA damage, and DNA repair are positively correlated with PLEK2 expression in LUAD cells (Jiang et al., 2020; Zhang et al., 2020; Wu et al., 2021; Zhou et al., 2022). Promoter hypomethylation may underlie its upregulation (Zhang et al., 2020). *In vitro*, FABP5 regulates fat metabolism by diverting fat into complex lipid synthesis instead of catabolism. FABP5 is also essential for cell cycle progression, migration, and tumor growth *in vivo* (Garcia et al., 2022). EC61γ has been shown to promote LUAD proliferation, metastasis, and invasion through the EGFR signaling pathway, suggesting this gene as a potential therapeutic target. Previous literature has indicated that the BEX family has diagnostic and prognostic value in LUAD, and that BEX4 is associated with clinicopathologic features, especially in higher-grade LUAD. LINC02535 has been shown to promote LUAD development through the NF-κB signaling pathway, and further pan-carcinoma analysis has demonstrated extensive prognostic value for LINC02535 in pan-carcinoma. While the mechanism of action of these genes in LUAD has been partially explored, their correlation with copper proliferation has not been investigated.

In the subsequent functional enrichment analysis, GO was used to analyze the differential genes of patients in the high-low risk group, which were mainly concentrated in chromosome-related signaling pathways, and studies have shown that copper ions can promote the formation of reactive oxygen species, which can damage DNA and chromatin (Garcia et al., 2022).The most significant pathways identified by KEGG analysis included cell cycle and DNA replication. The most significant pathways in HALLMARK enrichment analysis include G2M chekpoint. Studies have shown that copper oxide nanoparticles can reduce the activity of mouse embryonic fibroblasts and stop the cell cycle at G2M (Luo et al., 2014). The binding of copper and DNA bases has a concentration dependent tolerance relationship, which can reshape the integrity of DNA by affecting the structure of B-DNA, and affect the process of DNA replication and transcription (Govindaraju et al., 2013). These results further reveal the role of copper proliferation-related genes in LUAD and provide a new direction to explore the role of copper proliferation-related genes in LUAD in the future.

According to the immunocheckpoint analysis between high and low risk groups, the expression of PDCD1 CD274 LAG3 TNFRSF14 NRP1 was differentially expressed. Studies have shown that the variation of PDCD1 and CD274 genes regulates the risk and prognosis of LUAD and LUSC. The IMVIGOR210 analysis found that the lower the risk score, the better the treatment effect.

Subsequent stage-based stratified analysis found that patients in the high-risk group had worse prognosis, and the difference between the high-low risk groups was more significant in stage 3 and stage 4 patients. These results increased the application value of risk scores in LUAD. Subsequently, five algorithms were used to analyze the degree of immune cell infiltration between TCGA-LUAD high and low risk groups, and it was found that there were statistical differences in macrophages among the four algorithms, and the proportion of some types of macrophages was higher in high risk groups. Studies have shown that targeting macrophages in drug-resistant advanced LUAD is a therapeutic approach (Yin et al., 2023), and recent single-cell sequencing data further revealed that macrophages may play an important role in LUAD brain metastasis patients (Sun et al., 2022). These results suggest the research direction of copper proliferation-related genes in LUAD.

There are still some limitations in our study. First of all, we only described the genes related to copper proliferation through two publicly available data sets, which were not verified *in vitro* and *in vivo* experiments. Secondly, the accuracy of the prognostic model lacks the validation of multi-center data, which may limit the scope of the prognostic model. Subsequently, the predictive effects of immune checkpoints and immunotherapy still need to be further demonstrated in clinical trials. Finally, the effects of copper proliferation-related genes on chromatin and other signaling pathways still need further molecular biological experiments to prove.

## Conclusion

In conclusion, we first conducted a comprehensive analysis of copper proliferating genes in the LUAD landscape and found that prognostic models constructed with copper proliferation-related genes could predict OS, immune checkpoint, and immunotherapy in LUAD. Further pathway enrichment analysis revealed that copper proliferation-related genes may affect chromatin structure and DNA replication and translation, thus influencing cell cycle. Meanwhile, immune cell infiltration analysis revealed that macrophages may be the key immune cells in this process.

## Data Availability

The datasets presented in this study can be found in online repositories. The names of the repository/repositories and accession number(s) can be found in the article/Supplementary Material.
